# Does Stress Explain the Effects of Sexual/Gender Minority Status on Children’s Behavioral and Emotional Risk?

**DOI:** 10.31586/ojp.2025.6188

**Published:** 2025-09-18

**Authors:** Shervin Assari, Alexandra Donovan, John Ashley Pallera, Gandom Assari, Babak Najand, Kamiar Alaei, Arash Alaei

**Affiliations:** 1 Department of Psychiatry, Charles R Drew University of Medicine and Science, Los Angeles, CA, USA; 2 Department of Internal Medicine, Charles R Drew University of Medicine and Science, Los Angeles, CA, USA; 3 Department of Public Health, Charles R Drew University of Medicine and Science, Los Angeles, CA, USA; 4 Charles R Drew University of Medicine and Science, Los Angeles, CA, USA; 5 Mayfair High School, Lakewood, CA, USA; 6 Marginalization-related Diminished Returns Center, Los Angeles, CA, USA; 7 Department of Health Science, College of Health & Human Services, California State University Long Beach, Long Beach, CA, USA; 8 Center for Global Health, College of Health & Human Services, California State University Long Beach, Long Beach, CA, USA

**Keywords:** Children, Adolescents, Sexual/Gender Minority, Suicide Attempt, Depression, Trauma, Adverse Life Conditions

## Abstract

**Background:**

Sexual and gender minority (SGM) youth are at elevated risk for adverse mental health and substance use outcomes. Stressors such as family conflict, discrimination, and trauma have been suggested as possible mediators of these disparities.

**Aims:**

This study examined whether family conflict, discrimination, and trauma mediate the associations between SGM identity and adverse outcomes, including suicide attempt, major depressive disorder (MDD), nicotine use, and marijuana use.

**Methods:**

Participants were children from the Adolescent Brain Cognitive Development (ABCD) study. SGM identity was reported at baseline, while outcomes included past MDD and suicide attempts as well as future nicotine and marijuana use. Structural equation modeling (SEM) was used to test both direct and indirect pathways linking SGM identity to mental health and behavioral outcomes.

**Results:**

No significant mediation was found through family conflict, discrimination, or trauma. Instead, effects of SGM identity were primarily direct: SGM youth had higher odds of past suicide attempts and MDD, as well as future marijuana use, but not future nicotine use. Stressor variables, however, were independently associated with outcomes. Discrimination predicted all outcomes; trauma was positively associated with suicide, nicotine, and marijuana use but not MDD; and family conflict predicted all outcomes except MDD.

**Conclusion:**

Family conflict, discrimination, and trauma did not mediate SGM disparities in mental health and substance use, but each emerged as an independent predictor of risk. These findings highlight the complexity of mechanisms underlying SGM-related disparities and suggest the need for future research to explore additional pathways and contextual influences.

## 1. Introduction

Sexual and gender minority (SGM) youth experience a disproportionately high burden of adverse health outcomes compared to their non-SGM peers [1–5]. Previous research has consistently shown elevated rates of suicide attempts [6–8] and major depressive disorder (MDD) [9–11] among SGM adolescents, reflecting the toll of minority stress and structural inequities on mental health. Beyond mental health, substance use disparities are also evident, with SGM youth demonstrating higher likelihood of engaging in marijuana and nicotine use [12,13]. Together, these outcomes pose critical challenges to adolescent health and well-being, highlighting the importance of unraveling mechanisms that contribute to these disparities.

One possible explanation for these disparities lies in the role of social and environmental stressors [14–16]. Factors such as family conflict, discrimination, and trauma are known to negatively affect youth mental health and increase risk for substance use [17,18]. These stressors are also disproportionately experienced by SGM youth, raising the possibility that they may serve as mediating pathways linking SGM identity to adverse outcomes [19,20]. Mediation by these stressors would suggest that interventions reducing family conflict, preventing discrimination, or addressing trauma exposure could mitigate the elevated risks observed among SGM adolescents.

Very few studies have tested mediators of the link between SGM and poor mental and behavioral outcomes in youth and adults. Hatzenbuehler proposed a conceptual framework that synthesized key insights from prior research. The framework outlined that (a) sexual minority individuals experience elevated stress stemming from stigma; (b) this stigma-related stress increases emotional dysregulation, social and interpersonal difficulties, and maladaptive cognitive processes that heighten susceptibility to mental health problems; and (c) these mechanisms function as pathways connecting stigma-related stress to psychopathology in SGM populations. This proposed model would deepen understanding of how stigma harms mental health of SGM youth and guide the development of both clinical interventions and prevention efforts. Hatzenbuehler reviews considerable evidence supporting his framework, particularly for depression, anxiety, and alcohol use disorders [21].

Some empirical studies have tested various mediators of SGM disparities in mental and behavioral health. In one study, [11] data from 4,274 participants in the Avon Longitudinal Study of Parents and Children (ALSPAC) were analyzed to examine links between sexual orientation and later suicidal ideation and self-harm (SISH). Sexual orientation was reported at age 15, while past-year SISH was assessed at age 20. Potential mediators included self-esteem (measured at age 17), depressive symptoms (measured at age 18), and SGM identity (Childhood Gender Nonconformity, assessed at 30–57 months). Two significant mediation pathways were observed: one through self-esteem alone, and another through self-esteem followed by depressive symptoms. Low self-esteem and elevated depressive symptoms partly accounted for the increased risk of SISH observed among SGM youth [11]. A second cross-sectional study explored how psychosocial factors mediate the link between sexual orientation and suicidal ideation among young men in China. The first pathway showed that sexual orientation was indirectly associated with suicidal ideation through family support and depressive symptoms. The second pathway indicated an indirect association through support from friends, self-esteem, and depressive symptoms. Authors concluded that enhancing family and friend support as well as strengthening self-esteem may be promising intervention targets to reduce suicide risk among sexual minority men [22]. The third study applied longitudinal mediation models to examine whether victimization could help explain mental health disparities among sexual minority adolescents. The study measured victimization, depressive symptoms, and suicidality across two waves spaced six months apart. Mediation analyses showed that sexual minority-specific victimization significantly accounted for the association between sexual minority status and both depressive symptoms and suicidality [23]. These findings support Hatzenbuehler’s minority stress framework, suggesting that various types of stressors may contribute to the elevated risk of depression, suicide, and substance use among sexual minority adolescents.

The present study aimed to test whether three types of stress, namely family conflict, discrimination, and trauma, mediate the associations between SGM [24] and adverse outcomes, including suicide attempt, MDD, nicotine use, and marijuana use. Drawing on data from the Adolescent Brain Cognitive Development (ABCD) study [25–37], we examined both direct and indirect pathways using structural equation modeling (SEM) [38–43]. Testing mediation processes at such an early developmental stage is scientifically valuable because minority stress mechanisms are typically studied in adolescence or adulthood. By examining these pathways at ages 9–10, this study addresses a critical gap in the literature and helps clarify when and how disparities first begin to emerge. This approach allowed us to test whether disparities in mental health and substance use outcomes among SGM youth could be explained by these stressor variables, or whether the associations reflect direct effects of SGM identity beyond these mediators.

## 2. Methods

### 2.1. Study Design and Participants

Data for this study came from the Adolescent Brain Cognitive Development (ABCD) Study [25–37], a large, ongoing, prospective cohort of U.S. children recruited from 21 sites nationwide. The ABCD study began in 2016–2018 and enrolled nearly 12,000 children aged 9–10 years at baseline, with annual follow-ups assessing biological, behavioral, and social development. Details of study recruitment and procedures have been described elsewhere.

For the present analysis, we included participants with available baseline data on sexual and gender minority (SGM) identity and mediator variables (family conflict, discrimination, and trauma). Mental health and behavioral outcomes were reported at baseline (past major depressive disorder [MDD] and suicide attempt) and follow-up waves (future nicotine and marijuana use). Participants were included if they had valid data for at least one outcome, using listwise deletion within each model.

### 2.2. Measures

#### 2.2.1. Sexual and Gender Minority (SGM) Identity

At baseline, participants self-reported aspects of sexual orientation and gender identity. Following established approaches in adolescent health research, individuals were classified as SGM if they endorsed non-heterosexual attraction, non-heterosexual identity, or a gender identity differing from their sex assigned at birth. A dichotomous indicator was created (1 = SGM, 0 = non-SGM) [44].

#### 2.2.2. Mental Health and Substance Use Outcomes

We examined four outcomes reflecting both internalizing problems and health-risk behaviors. Suicide attempt: Lifetime history of suicide attempt was assessed using youth self-report at baseline [45,46]. Major depressive disorder (MDD): Past-year MDD diagnosis at baseline [47,48] was derived from the computerized Kiddie Schedule for Affective Disorders and Schizophrenia (KSADS-5) [49–51]. Nicotine use: Self-reported use of tobacco or nicotine products (e.g., e-cigarettes, combustible cigarettes) from baseline to the fourth yearly follow-up was coded as any use versus none [35,52–54]. Marijuana use: Self-reported marijuana use from baseline to the fourth yearly follow-up was similarly coded as any use versus none [35,52–54].

#### 2.2.3. Mediator Variables

We focused on three stressor variables commonly hypothesized as mechanisms in minority stress theory [19–21]. Family conflict: Assessed using items from the Family Environment Scale (FES). Items capture the frequency of quarrels, hostility, and lack of family cohesion. Scores were standardized for analysis [55]. Discrimination: Measured using the Perceived Discrimination Scale adapted for adolescents. Items ask about experiences of unfair treatment due to race/ethnicity, gender, or other social identities [56]. Higher scores reflect greater exposure to discrimination. Trauma exposure: Parent reported exposure to events such as physical assault, witnessing violence, or serious accidents on the KSADS-PTSD module, and a total trauma score was created [57].

#### 2.2.4. Covariates

Models adjusted for demographic and socioeconomic covariates, including age, sex at birth, parental education, and household income, to account for confounding in the associations of SGM identity with outcomes.

### 2.3. Statistical Analysis

Analyses were conducted using structural equation modeling (SEM) [38,40,42,58–60] in Stata version 18.0. We specified a path model in which SGM identity predicted each outcome directly, as well as indirectly through family conflict, discrimination, and trauma. Each mediator was modeled simultaneously to estimate unique indirect effects. Significance of indirect effects was evaluated using bias-corrected bootstrapped confidence intervals (10,000 resamples). All models accounted for clustering within study sites and used robust standard errors to address non-normality. Model fit was evaluated with multiple indices, including the root mean square error of approximation (RMSEA < 0.08 indicating acceptable fit), comparative fit index (CFI > 0.90), and standardized root mean square residual (SRMR < 0.08).

### 2.4. Ethical Considerations

The ABCD study received approval from the Institutional Review Board (IRB) at the University of California, San Diego, and all participating sites obtained local IRB approvals. Parents or guardians provided written informed consent, and children provided assent prior to data collection. The present study was conducted under the ABCD Data Use Agreement, and the analyses were exempt from additional IRB review at our institution because they used fully de-identified, publicly available data.

## 3. Results

### 3.1. Descriptive Data

Table 1 summarizes the descriptive statistics for the continuous variables in the study. The average age of participants was 9.48 months (SE = 0.00, 95% CI [9.47, 9.49]). Parents reported a relatively high level of educational attainment, with the mean parental education corresponding to 16.71 years (SE = 0.03, 95% CI [16.66, 16.76]). With respect to psychosocial indicators, participants reported an average trauma score of 0.52 (SE = 0.01, 95% CI [0.50, 0.54]) and a mean discrimination score of 1.20 (SE = 0.00, 95% CI [1.19, 1.21]). Family conflict was reported at a mean of 0.72 (SE = 0.01, 95% CI [0.70, 0.74]).

Table 2 summarizes the categorical characteristics of the study sample. Only a small proportion of participants identified as SGM (1.50%). Slightly more participants were male (52.14%) than female (47.86%). Most participants lived in married households (67.30%). Participants showed low rates of lifetime suicide attempt (2.59%) and past year diagnosis of major depressive disorder (MDD, 2.15%). About 5.28% of participants had reported nicotine use and 3.41% reported marijuana use by the fourth yearly follow up.

### 3.2. SEM Results

As shown in Table 3, SEM results indicated that SGM youth had higher risk of suicide attempt, MDD, and marijuana use, but not nicotine use. Stressors such as family conflict, discrimination, and trauma independently predicted outcomes but did not serve as mediators of SGM disparities.

#### 3.2.1. Suicide Attempts (Lifetime)

Higher family conflict was significantly associated with greater odds of reporting a past suicide attempt (b = 0.009, 95% CI [0.005, 0.014], p < .001). Discrimination was also a robust predictor of suicide attempt (b = 0.032, 95% CI [0.021, 0.043], p < .001). Trauma exposure showed a marginal association (b = 0.004, 95% CI [0.000, 0.007], p = .067). Being an SGM youth was positively associated with suicide attempt (b = 0.040, 95% CI [0.005, 0.074], p = .024). Living in a married household was negatively associated with suicide attempt (b = −0.024, 95% CI [−0.034, −0.013], p < .001).

#### 3.2.2. Major Depressive Disorder (Past-year diagnosis)

Discrimination was positively associated with past-year MDD (b = 0.029, 95% CI [0.022, 0.036], p < .001). Family conflict and trauma exposure were not significantly related to MDD (both p > .10). SGM youth showed higher odds of MDD (b = 0.035, 95% CI [0.010, 0.060], p = .007). Older age was also positively related to MDD (b = 0.006, 95% CI [0.000, 0.012], p = .050).

#### 3.2.3. Nicotine Use (Future)

Family conflict predicted greater odds of future nicotine use (b = 0.006, 95% CI [0.002, 0.010], p = .005), as did discrimination (b = 0.020, 95% CI [0.011, 0.030], p < .001) and trauma exposure (b = 0.007, 95% CI [0.003, 0.011], p = .001). SGM status was not significantly related to future nicotine use (b = 0.023, 95% CI [–0.010, 0.056], p = .172). Older age was strongly associated with nicotine use (b = 0.033, 95% CI [0.025, 0.041], p < .001). Males showed marginally lower nicotine use compared with females (b = −0.008, 95% CI [–0.016, 0.000], p = .058).

#### 3.2.4. Marijuana Use (Future)

Family conflict predicted greater odds of future marijuana use (b = 0.003, 95% CI [0.000, 0.007], p = .036). Discrimination was also a significant predictor (b = 0.014, 95% CI [0.006, 0.022], p < .001), as was trauma exposure (b = 0.004, 95% CI [0.001, 0.007], p = .015). SGM youth had elevated odds of marijuana use (b = 0.042, 95% CI [0.015, 0.069], p = .002). Older age was associated with increased odds of marijuana use (b = 0.028, 95% CI [0.022, 0.034], p < .001).

#### 3.2.5. Association with Mediators

In models describing covariate and SGM relationships to the mediators, SGM identity was not significantly related to family conflict (b = −0.085, 95% CI [−0.235, 0.064], p = .263), discrimination (b = 0.002, 95% CI [−0.064, 0.068], p = .950), or trauma exposure (b = 0.099, 95% CI [−0.060, 0.259], p = .221). However, several covariates were associated with mediator levels. Male sex was positively related to family conflict (b = 0.082, 95% CI [0.046, 0.119], p < .001) and discrimination (b = 0.067, 95% CI [0.051, 0.083], p < .001). Higher parental education was negatively associated with family conflict (b = −0.012, 95% CI [−0.019, −0.004], p = .001) and discrimination (b = −0.022, 95% CI [−0.025, −0.019], p < .001). Living in a married household was inversely associated with family conflict (b = −0.167, 95% CI [−0.208, −0.125], p < .001), discrimination (b = −0.079, 95% CI [−0.098, −0.061], p < .001), and trauma exposure (b = −0.275, 95% CI [−0.319, −0.231], p < .001).

**Figure 1.**
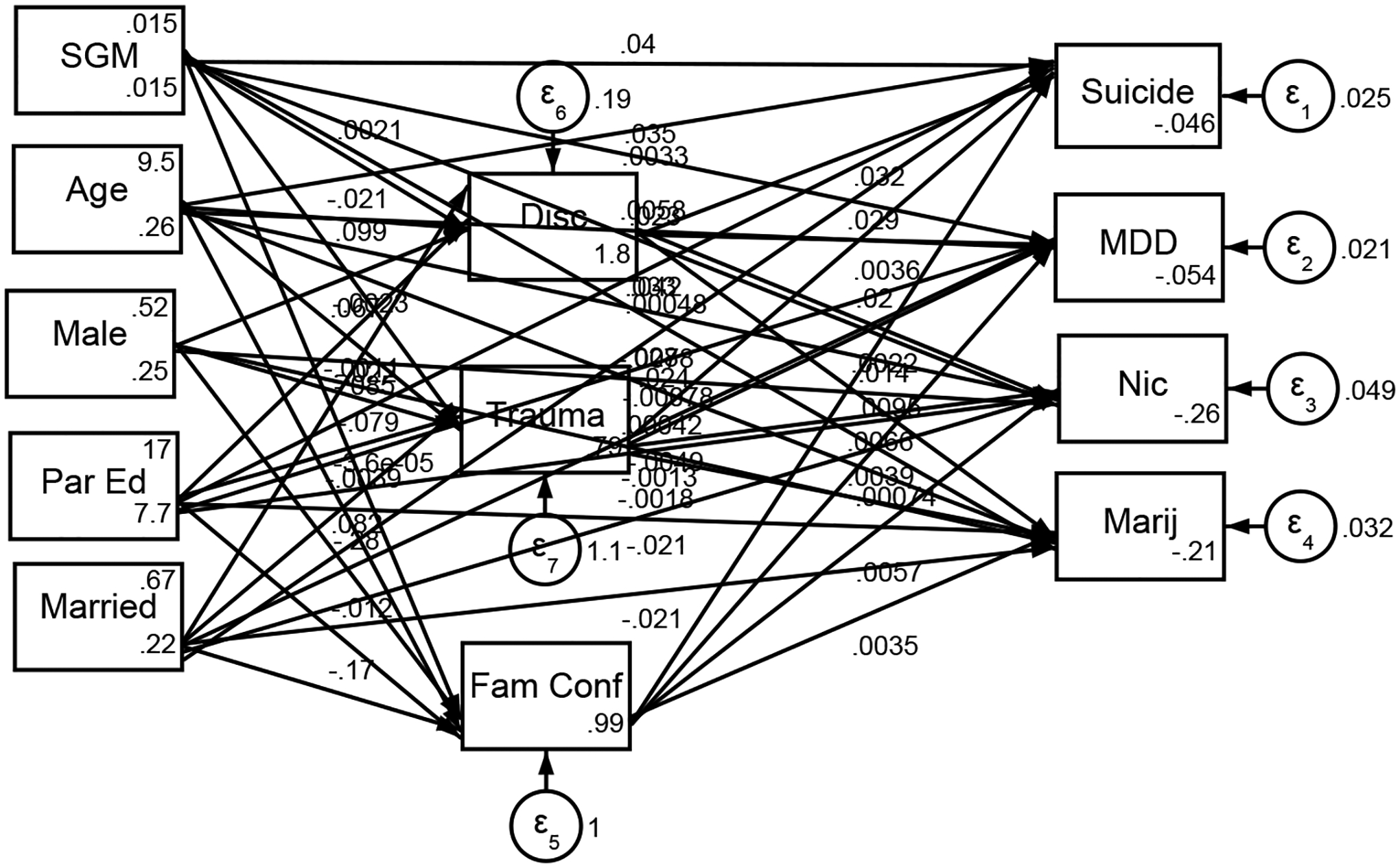
Structural Equation Model (***Note*:** Par Ed= Parental Education; SGM = Sexual and Gender Minority; Marij = Marijuana Use; Nic= Nicotine Use; MDD= Major Depressive Disorder; Suicide= Suicide Attempt; Disc= Discrimination; Trauma = Number of Traumatic Events; Fam Conf= Family Conflict)

Figure 1 presents the structural equation model illustrating that the associations between the independent and dependent variables are not explained by the proposed mediator. The path analysis indicates that the mediator does not carry or reduce the effect; instead, the relationship remains direct and significant. This suggests that the predictor influences the outcome without being channeled through the intermediary variable, highlighting the absence of mediation in the model.

## 4. Discussion

Using structural equation modeling (SEM) with baseline ABCD Study data, we examined how sexual and gender minority (SGM) identity, family stressors, and adverse social experiences shaped risk for suicide attempt, major depressive disorder (MDD), and future nicotine and marijuana use in early adolescence. Our results extend prior work by showing that SGM youth already exhibit significant disparities in mental health at ages 9–10, a developmental stage often considered early for the emergence of such risks. Specifically, SGM youth demonstrated significantly higher odds of suicide attempt, MDD, and future marijuana use compared with their non-SGM peers, though no such disparity was observed for future nicotine use.

Beyond SGM identity, stressors such as family conflict, discrimination, and trauma exposure emerged as robust independent predictors of adverse outcomes. Each was significantly associated with suicide attempts and future substance use, and discrimination in particular was strongly related to both suicide attempts and MDD. However, these stressors did not act as mediators of the disparities between SGM and non-SGM youth. This suggests that while adversity worsens outcomes across the entire sample, the elevated risks faced by SGM youth cannot be explained by greater exposure to family conflict, discrimination, or trauma alone. Rather, the risks appear to operate independently, consistent with processes unique to minority stress.

Our findings that SGM youth were more likely to report a past suicide attempt and past-year MDD align with a growing body of evidence on disparities in psychiatric outcomes among SGM populations. Minority stress theory provides a compelling framework for understanding these results, highlighting how stigma, exclusion, and structural disadvantage produce chronic stressors that accumulate over time to harm mental health. The fact that SGM disparities remained even after adjusting for family conflict, discrimination, and trauma suggests that other unmeasured factors may be contributing to this vulnerability. These could include internalized stigma, concealment of one’s identity, or victimization specifically tied to sexual and gender identity. Moreover, given the young age of the ABCD sample, these disparities may represent early manifestations of vulnerabilities that widen across adolescence and into adulthood if left unaddressed.

One of the most notable distinctions in our findings was that SGM identity predicted marijuana use but not nicotine use. This difference may reflect shifts in substance use patterns among contemporary adolescents. While tobacco prevention campaigns and policy restrictions have led to historically low rates of combustible cigarette use in youth, marijuana remains widely available and increasingly normalized in the context of legalization in many U.S. states. For SGM youth, marijuana may serve as a coping strategy for stress and stigma, consistent with self-medication frameworks [61]. Prior research has shown higher marijuana use among SGM populations in adolescence and adulthood, often linked to efforts to manage minority stress [62]. The absence of an SGM disparity in nicotine use could indicate both the success of tobacco control measures and the fact that SGM youth may be drawn to substances that are perceived as more socially acceptable among peers.

Our results further highlight the independent role of adversity in shaping mental health and substance use outcomes. Family conflict was consistently associated with suicide attempt, nicotine use, and marijuana use, while discrimination was strongly associated with suicide attempt and MDD. Trauma exposure also increased risk for substance use. These findings are consistent with decades of research documenting the harmful impact of family dysfunction and social marginalization on youth development. However, the absence of mediation indicates that such stressors do not fully explain disparities for SGM youth. In other words, while reducing family conflict, discrimination, and trauma would likely improve outcomes for all youth, these strategies alone may not eliminate the disproportionate risks faced by SGM populations. This underscores the importance of interventions that address minority-specific processes, such as identity-related stress and peer victimization.

Our analyses also identified protective factors. Living in a married household was associated with lower odds of suicide attempt and lower reported levels of family conflict, discrimination, and trauma. Similarly, higher parental education was associated with reduced family conflict and discrimination. These findings emphasize the role of stable family structures and socioeconomic resources as buffers against psychosocial stress and adverse outcomes. While these factors did not eliminate SGM disparities, they highlight potential points of intervention. Supporting families through education, strengthening economic resources, and promoting family cohesion may foster resilience, even among youth facing identity-related risks.

### 4.1. Implications for Prevention and Policy

The findings carry important implications for prevention and policy. First, suicide prevention strategies for adolescents should explicitly recognize the heightened vulnerability of SGM youth. Programs designed for general populations may not adequately address minority stress processes; tailored approaches are needed to reduce suicide risk and promote mental health in these groups. Second, interventions that target family functioning and reduce exposure to discrimination could yield broad benefits, particularly when combined with programs aimed at fostering affirming environments for SGM youth. Finally, substance use prevention efforts should adapt to shifting patterns of risk, with increased attention to marijuana use as a salient concern for SGM adolescents.

### 4.2. Limitations

Several limitations should be acknowledged. The analyses were cross-sectional, limiting the ability to infer causal pathways and the potential for mediators to influence outcomes. Longitudinal follow-up of the ABCD cohort will be critical to examine how disparities evolve across adolescence and into young adulthood. In addition, measures of family conflict, discrimination, and trauma were based on self- and parent-report, which may be subject to reporting bias. The relatively small proportion of SGM youth, while consistent with population prevalence at this age, limited statistical power and may have produced conservative estimates of disparities. Lack of inclusion of race/ethnicity as a covariate is important. Race/ethnicity likely impacts measures of discrimination, substance use, and mental health. Also, race/ethnic minority youth may have added cultural stigma on belonging to SGM [63,64]. It is important to note that our findings may actually underestimate the true extent of SGM disparities. Sexual orientation and gender identity often consolidate later in adolescence, and prior work suggests that disparities in mental health and substance use widen with age. Thus, the current results likely represent conservative estimates of these differences. Finally, the measures of future nicotine and marijuana use were based on self-reports of low-level use, which may not fully capture actual trajectories of substance use as participants age or separate normative experimental behaviors from problematic use.

### 4.3. Future Directions

Future research should build on these findings in several important ways. Longitudinal analyses of the ABCD cohort will allow researchers to test developmental trajectories, clarifying whether early disparities among SGM youth widen, narrow, or shift as adolescents mature. Such work could examine whether early marijuana use predicts later mental health challenges or interacts with minority stress processes to shape long-term outcomes. Additionally, integrating peer relationships and school climate into future models could help identify how affirming or hostile social environments impact risk. Neuroimaging data available in ABCD also provides an opportunity to examine biological stress pathways and neural mechanisms of risk and resilience among SGM youth, which may offer further insight into the unique vulnerabilities identified here. Finally, intervention research is needed to test programs that directly address minority stress—such as school-based affirming policies, peer support groups, or family-based acceptance interventions—and to evaluate whether these approaches reduce disparities in suicide attempts, depression, and substance use.

## 5. Conclusion

In conclusion, this study demonstrates that SGM youth face disproportionately high risks of suicide attempt, MDD, and marijuana use in early adolescence, risks that cannot be fully explained by exposure to family conflict, discrimination, or trauma. Although these contextual stressors were strong predictors of mental health and substance use for all youth, they did not mediate SGM disparities. These findings suggest that minority stress processes operate through unique pathways. Prevention strategies should combine efforts to reduce contextual stressors with targeted approaches that affirm SGM identities, reduce stigma, and provide culturally responsive support. Broad structural efforts to reduce discrimination and strengthen family and socioeconomic support systems are crucial for reducing disparities and fostering resilience within this vulnerable population.

## Figures and Tables

**Table 1.** Descriptive Data, Continuous Variables

	Mean	Std. Err.	95% CI	
Age (Years)	9.48	0.00	9.47	9.49
Parental Education (Years)	16.71	0.03	16.66	16.76
Trauma (n)	0.52	0.01	0.50	0.54
Perceived Discrimination	1.20	0.00	1.19	1.21
Family Conflict	0.72	0.01	0.70	0.74

**Table 2.** Descriptive Data, Categorical Variables

	Freq.	Percent
Gender/Sex Minority		
No	11,671	98.50
Yes	178	1.50
Sex/Gender		
Female	5,673	47.86
Male	6,181	52.14
Married Family		
Unmarried	3,877	32.70
Married	7,980	67.30
Suicide Attempt History (Lifetime)		
No	4,583	97.41
Yes	122	2.59
MDD (Past-year diagnosis)		
No	9,146	97.85
Yes	201	2.15
Future Nicotine Use		
No	11,097	94.72
Yes	618	5.28
Future Marijuana Use		
No	11,315	96.59
Yes	400	3.41

***Note:*** Major Depressive Disorder (MDD)

**Table 3.** Summary of the Structural Equation Model Findings

	Coefficient	Std. Err.	95%	CI	p
Suicide Attempt History (Lifetime)					
Family Conflict	0.009	0.002	0.005	0.014	< 0.001
Discrimination	0.032	0.006	0.021	0.043	< 0.001
Trauma	0.004	0.002	0.000	0.007	0.067
Sexual/Gender Minority	0.040	0.018	0.005	0.074	0.024
Age	0.003	0.005	−0.006	0.012	0.468
Parental Education (years)	0.000	0.001	−0.001	0.002	0.599
Married household	−0.024	0.005	−0.034	−0.013	< 0.001
					
MDD (Past-year diagnosis)					
Family Conflict	0.001	0.001	−0.002	0.004	0.616
Discrimination	0.029	0.003	0.022	0.036	< 0.001
Trauma	0.002	0.001	−0.001	0.005	0.144
Sexual/Gender Minority	0.035	0.013	0.010	0.060	0.007
Age	0.006	0.003	0.000	0.012	0.050
Parental Education (years)	−0.001	0.001	−0.002	0.000	0.175
Married household	−0.005	0.003	−0.012	0.002	0.153
					
Future Nicotine Use					
Family Conflict	0.006	0.002	0.002	0.010	0.005
Discrimination	0.020	0.005	0.011	0.030	< 0.001
Trauma	0.007	0.002	0.003	0.011	0.001
Sexual/Gender Minority	0.023	0.017	−0.010	0.056	0.172
Age	0.033	0.004	0.025	0.041	< 0.001
Male	−0.008	0.004	−0.016	0.000	0.058
Parental Education (years)	−0.001	0.001	−0.003	0.000	0.094
Married household	−0.021	0.005	−0.030	−0.012	< 0.001
					
Future Marijuana Use					
Family Conflict	0.003	0.002	0.000	0.007	0.036
Discrimination	0.014	0.004	0.006	0.022	< 0.001
Trauma	0.004	0.002	0.001	0.007	0.015
Sexual/Gender Minority	0.042	0.014	0.015	0.069	0.002
Age	0.028	0.003	0.022	0.034	0.000
Male	0.000	0.003	−0.006	0.007	0.901
Parental Education (years)	−0.002	0.001	−0.003	−0.001	0.006
Married household	−0.021	0.004	−0.028	−0.013	< 0.001
					
Family Conflict					
Sexual/Gender Minority	−0.085	0.076	−0.235	0.064	0.263
Age	0.000	0.018	−0.036	0.036	0.998
Male	0.082	0.019	0.046	0.119	< 0.001
Parental Education (years)	−0.012	0.004	−0.019	−0.004	0.001
Married household	−0.167	0.021	−0.208	−0.125	< 0.001
					
Discrimination					
Sexual/Gender Minority	0.002	0.034	−0.064	0.068	0.950
Age	−0.021	0.008	−0.037	−0.005	0.010
Male	0.067	0.008	0.051	0.083	< 0.001
Parental Education (years)	−0.022	0.002	−0.025	−0.019	< 0.001
Married household	−0.079	0.009	−0.098	−0.061	< 0.001
					
Trauma					
Sexual/Gender Minority	0.099	0.081	−0.060	0.259	0.221
Age	−0.002	0.019	−0.040	0.036	0.907
Male	−0.001	0.020	−0.040	0.038	0.957
Parental Education (years)	−0.004	0.004	−0.011	0.004	0.307
Married household	−0.275	0.022	−0.319	−0.231	< 0.001

***Note:*** Major Depressive Disorder (MDD)

## References

[1] Yıldız E Suicide in sexual minority populations: A systematic review of evidence-based studies. Arch Psychiatr Nurs 2018, 32, 650–659, doi:10.1016/j.apnu.2018.03.003. 30029759

[2] Kittiteerasack P; Matthews AK; Steffen A; Corte C; McCreary LL; Bostwick W; Park C; Johnson TP The influence of minority stress on indicators of suicidality among lesbian, gay, bisexual and transgender adults in Thailand. J Psychiatr Ment Health Nurs 2021, 28, 656–669, doi:10.1111/jpm.12713. 33190351

[3] Bränström R; van der Star A; Pachankis JE Untethered lives: barriers to societal integration as predictors of the sexual orientation disparity in suicidality. Soc Psychiatry Psychiatr Epidemiol 2020, 55, 89–99, doi:10.1007/s00127-019-01742-6. 31300892

[4] Pitoňák M; Potočár L; Formánek T Mental health and help-seeking in Czech sexual minorities: a nationally representative cross-sectional study. Epidemiol Psychiatr Sci 2024, 33, e16, doi:10.1017/s2045796024000210. 38511544 PMC11022263

[5] Almazan EP; Roettger ME; Acosta PS Measures of sexual minority status and suicide risk among young adults in the United States. Arch Suicide Res 2014, 18, 274–281, doi:10.1080/13811118.2013.824832. 24611686

[6] Horwitz AG; Berona J; Busby DR; Eisenberg D; Zheng K; Pistorello J; Albucher R; Coryell W; Favorite T; Walloch JC; Variation in Suicide Risk among Subgroups of Sexual and Gender Minority College Students. Suicide Life Threat Behav 2020, 50, 1041–1053, doi:10.1111/sltb.12637. 32291833 PMC7981781

[7] Hill AO; Lyons A; Power J; Amos N; Ferlatte O; Jones J; Carman M; Bourne A Suicidal Ideation and Suicide Attempts Among Lesbian, Gay, Bisexual, Pansexual, Queer, and Asexual Youth: Differential Impacts of Sexual Orientation, Verbal, Physical, or Sexual Harassment or Assault, Conversion Practices, Family or Household Religiosity, and School Experience. LGBT Health 2022, 9, 313–324, doi:10.1089/lgbt.2021.0270. 35420458

[8] Hill AO; Kaneko N; Amos N; Bourne A; Imahashi M; Armstrong G; Gilmour S High suicidality rates among LGB+ youth in Japan: Demographic and psychosocial correlates. J Affect Disord 2025, 387, 119468, doi:10.1016/j.jad.2025.119468. 40447151

[9] Lucassen MF; Stasiak K; Samra R; Frampton CM; Merry SN Sexual minority youth and depressive symptoms or depressive disorder: A systematic review and meta-analysis of population-based studies. Aust N Z J Psychiatry 2017, 51, 774–787, doi:10.1177/0004867417713664. 28565925

[10] Long Y; Pegg S; Bean CAL; Kittleson A; Clark K; Kujawa A Depressive symptoms and suicidal ideation in sexual minority adolescents: An examination of social reward responsiveness and support as moderators. J Mood Anxiety Disord 2024, 8, doi:10.1016/j.xjmad.2024.100090. PMC11719950 39803365

[11] Oginni OA; Robinson EJ; Jones A; Rahman Q; Rimes KA Mediators of increased self-harm and suicidal ideation in sexual minority youth: a longitudinal study. Psychol Med 2019, 49, 2524–2532, doi:10.1017/s003329171800346x. 30468143

[12] Schilt-Solberg MA; Blair LM; Kurzer J Intersectionality in substance use disorders: Examining gender, race/ethnicity, and sexual orientation in the 2021–2022 National Survey on Drug Use and Health. Addict Behav Rep 2025, 21, 100587, doi:10.1016/j.abrep.2025.100587. 40524897 PMC12169232

[13] Jun HJ; Webb-Morgan M; Felner JK; Wisdom JP; Haley SJ; Austin SB; Katuska LM; Corliss HL Sexual orientation and gender identity disparities in substance use disorders during young adulthood in a United States longitudinal cohort. Drug Alcohol Depend 2019, 205, 107619, doi:10.1016/j.drugalcdep.2019.107619. 31678835 PMC7437659

[14] Chaudoir SR; Wang K; Pachankis JE What reduces sexual minority stress? A review of the intervention “toolkit”. J Soc Issues 2017, 73, 586–617, doi:10.1111/josi.12233. 29170566 PMC5695701

[15] Bränström R Minority stress factors as mediators of sexual orientation disparities in mental health treatment: a longitudinal population-based study. J Epidemiol Community Health 2017, 71, 446–452, doi:10.1136/jech-2016-207943. 28043996 PMC5484026

[16] McConnell EA; Janulis P; Phillips G 2nd; Truong R; Birkett M Multiple Minority Stress and LGBT Community Resilience among Sexual Minority Men. Psychol Sex Orientat Gend Divers 2018, 5, 1–12, doi:10.1037/sgd0000265. 29546228 PMC5846479

[17] Amaro H; Sanchez M; Bautista T; Cox R Social vulnerabilities for substance use: Stressors, socially toxic environments, and discrimination and racism. Neuropharmacology 2021, 188, 108518, doi:10.1016/j.neuropharm.2021.108518. 33716076 PMC8126433

[18] Sinha R Stress and substance use disorders: risk, relapse, and treatment outcomes. J Clin Invest 2024, 134, doi:10.1172/jci172883. PMC11324296 39145454

[19] Trujillo MA; Newman DB; Mendes WB Sexual orientation and daily stress and well-being. Health Psychol 2025, 44, 197–206, doi:10.1037/hea0001460. 39992765 PMC12263077

[20] Jabson JM; Farmer GW; Bowen DJ Stress mediates the relationship between sexual orientation and behavioral risk disparities. BMC Public Health 2014, 14, 401, doi:10.1186/1471-2458-14-401. 24767172 PMC4038400

[21] Hatzenbuehler ML How does sexual minority stigma “get under the skin”? A psychological mediation framework. Psychol Bull 2009, 135, 707–730, doi:10.1037/a0016441. 19702379 PMC2789474

[22] Huang Y; Liu J; Huang G; Zhu D; Zhou Y; Hu J Understanding suicidal ideation disparity between sexual minority and heterosexual Chinese young men: a multiple mediation model of social support sources, self-esteem, and depressive symptoms. Front Psychiatry 2024, 15, 1265722, doi:10.3389/fpsyt.2024.1265722. 38559394 PMC10978729

[23] Burton CM; Marshal MP; Chisolm DJ; Sucato GS; Friedman MS Sexual minority-related victimization as a mediator of mental health disparities in sexual minority youth: a longitudinal analysis. J Youth Adolesc 2013, 42, 394–402, doi:10.1007/s10964-012-9901-5. 23292751 PMC3570607

[24] Potter AS; Dube SL; Barrios LC; Bookheimer S; Espinoza A; Feldstein Ewing SW; Freedman EG; Hoffman EA; Ivanova M; Jefferys H; Measurement of gender and sexuality in the Adolescent Brain Cognitive Development (ABCD) study. Dev Cogn Neurosci 2022, 53, 101057, doi:10.1016/j.dcn.2022.101057. 35026661 PMC8759998

[25] Alcohol Research: Current Reviews Editorial, S. NIH’s Adolescent Brain Cognitive Development (ABCD) Study. Alcohol Res 2018, 39, 97. 30557152 10.35946/arcr.v39.1.12 PMC6104964

[26] Casey B; Cannonier T; Conley MI; Cohen AO; Barch DM; Heitzeg MM; Soules ME; Teslovich T; Dellarco DV; Garavan H The adolescent brain cognitive development (ABCD) study: imaging acquisition across 21 sites. Developmental cognitive neuroscience 2018, 32, 43–54. 29567376 10.1016/j.dcn.2018.03.001 PMC5999559

[27] Casey BJ; Cannonier T; Conley MI; Cohen AO; Barch DM; Heitzeg MM; Soules ME; Teslovich T; Dellarco DV; Garavan H; The Adolescent Brain Cognitive Development (ABCD) study: Imaging acquisition across 21 sites. Dev Cogn Neurosci 2018, 32, 43–54, doi:10.1016/j.dcn.2018.03.001. 29567376 PMC5999559

[28] Cetin-Karayumak S; Zhang F; Billah T; Zekelman L; Makris N; Pieper S; O’Donnell LJ; Rathi Y Harmonized diffusion MRI data and white matter measures from the Adolescent Brain Cognitive Development Study. bioRxiv 2023, doi:10.1101/2023.04.04.535587. PMC10899197 38413633

[29] Compton WM; Dowling GJ; Garavan H Ensuring the Best Use of Data: The Adolescent Brain Cognitive Development Study. JAMA Pediatrics 2019, 173, 809–810, doi:10.1001/jamapediatrics.2019.2081. 31305867 PMC8056387

[30] Dick AS; Watts AL; Heeringa S; Lopez DA; Bartsch H; Fan CC; Palmer C; Reuter C; Marshall A; Haist F; Meaningful Effects in the Adolescent Brain Cognitive Development Study. bioRxiv 2020, 2020.2009.2001.276451, doi:10.1101/2020.09.01.276451.

[31] Ewing SWF; Dash GF; Thompson WK; Reuter C; Diaz VG; Anokhin A; Chang L; Cottler LB; Dowling GJ; LeBlanc K Measuring retention within the adolescent brain cognitive development (ABCD) SM study. Developmental cognitive neuroscience 2022, 54, 101081. 35152002 10.1016/j.dcn.2022.101081 PMC8844713

[32] Gray JC; Schvey NA; Tanofsky-Kraff M Demographic, psychological, behavioral, and cognitive correlates of BMI in youth: Findings from the Adolescent Brain Cognitive Development (ABCD) study. Psychol Med 2019, 50, 1–9, doi:10.1017/S0033291719001545. 31288867

[33] Hagler DJ Jr.; Hatton S; Cornejo MD; Makowski C; Fair DA; Dick AS; Sutherland MT; Casey BJ; Barch DM; Harms MP; Image processing and analysis methods for the Adolescent Brain Cognitive Development Study. NeuroImage 2019, 202, doi:10.1016/j.neuroimage.2019.116091. 31415884 PMC6981278

[34] Lisdahl KM; Sher KJ; Conway KP; Gonzalez R; Ewing SWF; Nixon SJ; Tapert S; Bartsch H; Goldstein RZ; Heitzeg M Adolescent brain cognitive development (ABCD) study: Overview of substance use assessment methods. Developmental cognitive neuroscience 2018, 32, 80–96, doi:10.1016/j.dcn.2018.02.007. 29559216 PMC6375310

[35] Lisdahl KM; Sher KJ; Conway KP; Gonzalez R; Feldstein Ewing SW; Nixon SJ; Tapert S; Bartsch H; Goldstein RZ; Heitzeg M Adolescent brain cognitive development (ABCD) study: Overview of substance use assessment methods. Dev Cogn Neurosci 2018, 32, 80–96, doi:10.1016/j.dcn.2018.02.007. 29559216 PMC6375310

[36] Luciana M; Bjork JM; Nagel BJ; Barch DM; Gonzalez R; Nixon SJ; Banich MT Adolescent neurocognitive development and impacts of substance use: Overview of the adolescent brain cognitive development (ABCD) baseline neurocognition battery. Dev Cogn Neurosci 2018, 32, 67–79, doi:10.1016/j.dcn.2018.02.006. 29525452 PMC6039970

[37] Luciana M; Bjork JM; Nagel BJ; Barch DM; Gonzalez R; Nixon SJ; Banich MT Adolescent neurocognitive development and impacts of substance use: Overview of the adolescent brain cognitive development (ABCD) baseline neurocognition battery. Developmental cognitive neuroscience 2018, 32, 67–79. 29525452 10.1016/j.dcn.2018.02.006 PMC6039970

[38] Bowen NK; Guo S Structural equation modeling; Oxford University Press: 2011.

[39] De Stavola BL; Daniel RM; Ploubidis GB; Micali N Mediation analysis with intermediate confounding: structural equation modeling viewed through the causal inference lens. Am J Epidemiol 2015, 181, 64–80, doi:10.1093/aje/kwu239. 25504026 PMC4383385

[40] Gunzler D; Chen T; Wu P; Zhang H Introduction to mediation analysis with structural equation modeling. Shanghai Arch Psychiatry 2013, 25, 390–394, doi:10.3969/j.issn.1002-0829.2013.06.009. 24991183 PMC4054581

[41] Leth-Steensen C; Gallitto E Testing Mediation in Structural Equation Modeling: The Effectiveness of the Test of Joint Significance. Educ Psychol Meas 2016, 76, 339–351, doi:10.1177/0013164415593777. 29795869 PMC5965588

[42] MacKinnon DP; Valente MJ Mediation from multilevel to structural equation modeling. Ann Nutr Metab 2014, 65, 198–204, doi:10.1159/000362505. 25413658 PMC4836377

[43] Olinsky A; Chen S; Harlow L The comparative efficacy of imputation methods for missing data in structural equation modeling. European Journal of Operational Research 2003, 151, 53–79.

[44] Liu S; English D; Xiao Y; Li Y; Niu L Sexual and gender minority identity, peer victimization, and suicidality in adolescents: A mediation study using the ABCD Study. J Child Psychol Psychiatry 2025, doi:10.1111/jcpp.14155. PMC12353512 40125918

[45] Grassia M; Gibb BE Rumination and Lifetime History of Suicide Attempts. Int J Cogn Ther 2009, 2, 400–406, doi:10.1521/ijct.2009.2.4.400. 31929850 PMC6953990

[46] Mitchell SM; Brown SL; Scanlon F; Swogger MT; Delgado D; Ventura MI; Bolaños AD; Morgan RD Lifetime History of Suicide Attempts among Not Guilty by Reason of Insanity State Hospital Inpatients: The Roles of past Harmful Substance Use and Current Social Support. Int J Forensic Ment Health 2020, 19, 341–353, doi:10.1080/14999013.2020.1775326. 33223964 PMC7678914

[47] Esfand SM; Null KE; Duda JM; de Leeuw J; Pizzagalli DA Lifetime history of major depressive disorder is associated with decreased reward learning: Evidence from a novel online version of the probabilistic reward task. J Affect Disord 2024, 350, 1007–1015, doi:10.1016/j.jad.2024.01.133. 38278332

[48] Bains N; Abdijadid S Major Depressive Disorder. In StatPearls; StatPearls Publishing Copyright © 2025, StatPearls Publishing LLC.: Treasure Island (FL), 2025.

[49] Makino T; Suzuki F; Nishiyama T; Ishibashi S; Nakamichi H; Iida T; Shimada S; Tomari S; Imanari E; Higashi T; Psychometrics of the kiddie schedule for affective disorders and schizophrenia present and lifetime version for DSM-5 in Japanese outpatients. Int J Methods Psychiatr Res 2023, 32, e1957, doi:10.1002/mpr.1957. 36593592 PMC10699497

[50] Dun Y; Li QR; Yu H; Bai Y; Song Z; Lei C; Li HH; Gong J; Mo Y; Li Y; Reliability and validity of the Chinese version of the kiddie-schedule for affective disorders and schizophrenia-present and lifetime version DSM-5 (K-SADS-PL-C DSM-5). J Affect Disord 2022, 317, 72–78, doi:10.1016/j.jad.2022.08.062. 36029880

[51] de la Peña FR; Rosetti MF; Rodríguez-Delgado A; Villavicencio LR; Palacio JD; Montiel C; Mayer PA; Félix FJ; Larraguibel M; Viola L; Construct validity and parent-child agreement of the six new or modified disorders included in the Spanish version of the Kiddie Schedule for Affective Disorders and Schizophrenia present and Lifetime Version DSM-5 (K-SADS-PL-5). J Psychiatr Res 2018, 101, 28–33, doi:10.1016/j.jpsychires.2018.02.029. 29529472

[52] Lisdahl KM; Tapert S; Sher KJ; Gonzalez R; Nixon SJ; Ewing SWF; Conway KP; Wallace A; Sullivan R; Hatcher K Substance use patterns in 9–10 year olds: Baseline findings from the adolescent brain cognitive development (ABCD) study. Drug and alcohol dependence 2021, 227, 108946, doi:10.1016/j.drugalcdep.2021.108946. 34392051 PMC8833837

[53] Luciana M; Bjork J; Nagel B; Barch D; Gonzalez R; Nixon S; Banich M Adolescent neurocognitive development and impacts of substance use: Overview of the adolescent brain cognitive development (ABCD) baseline neurocognition battery. Developmental cognitive neuroscience 2018, 32, 67–79. 29525452 10.1016/j.dcn.2018.02.006 PMC6039970

[54] Sullivan RM; Wade NE; Wallace AL; Tapert SF; Pelham WE 3rd; Brown SA; Cloak CC; Feldstein Ewing SW; Madden PAF; Martz ME; Substance use patterns in 9 to 13-year-olds: Longitudinal findings from the Adolescent Brain Cognitive Development (ABCD) study. Drug Alcohol Depend Rep 2022, 5, doi:10.1016/j.dadr.2022.100120. PMC9850746 36687306

[55] Gonzalez R; Thompson EL; Sanchez M; Morris A; Gonzalez MR; Feldstein Ewing SW; Mason MJ; Arroyo J; Howlett K; Tapert SF; An update on the assessment of culture and environment in the ABCD Study^®^: Emerging literature and protocol updates over three measurement waves. Dev Cogn Neurosci 2021, 52, 101021, doi:10.1016/j.dcn.2021.101021. 34700197 PMC8551602

[56] Chen S; Lopez-Quintero C; Elton A Perceived Racism, Brain Development, and Internalizing and Externalizing Symptoms: Findings From the ABCD Study. J Am Acad Child Adolesc Psychiatry 2025, doi:10.1016/j.jaac.2025.04.005. PMC13014306 40222403

[57] Hoffman EA; Clark DB; Orendain N; Hudziak J; Squeglia LM; Dowling GJ Stress exposures, neurodevelopment and health measures in the ABCD study. Neurobiol Stress 2019, 10, 100157, doi:10.1016/j.ynstr.2019.100157. 30949565 PMC6430638

[58] Moerkerke B; Loeys T; Vansteelandt S Structural equation modeling versus marginal structural modeling for assessing mediation in the presence of posttreatment confounding. Psychol Methods 2015, 20, 204–220, doi:10.1037/a0036368. 25751514

[59] Ullman JB; Bentler PM Structural equation modeling. Handbook of psychology, second edition 2012, 2.

[60] West SG; Wu W; McNeish D; Savord A Model fit in structural equation modeling. Handbook of structural equation modeling 2023, 2, 184–205.

[61] Garland EL; Pettus-Davis C; Howard MO Self-medication among traumatized youth: structural equation modeling of pathways between trauma history, substance misuse, and psychological distress. J Behav Med 2013, 36, 175–185, doi:10.1007/s10865-012-9413-5. 22454227 PMC3466352

[62] Russell ST; Fish JN Sexual minority youth, social change, and health: A developmental collision. Res Hum Dev 2019, 16, 5–20, doi:10.1080/15427609.2018.1537772. 31602178 PMC6786797

[63] Layland EK; Carter JA; Perry NS; Cienfuegos-Szalay J; Nelson KM; Bonner CP; Rendina HJ A systematic review of stigma in sexual and gender minority health interventions. Transl Behav Med 2020, 10, 1200–1210, doi:10.1093/tbm/ibz200. 33044540 PMC7549413

[64] Swann G; Stephens J; Newcomb ME; Whitton SW Effects of sexual/gender minority- and race-based enacted stigma on mental health and substance use in female assigned at birth sexual minority youth. Cultur Divers Ethnic Minor Psychol 2020, 26, 239–249, doi:10.1037/cdp0000292. 31021146 PMC6814455

